# The New York Institute of Stomatology

**Published:** 1898-09

**Authors:** 

**Affiliations:** The New York Institute of Stomatology


### Reports of Society Meetings.

### THE NEW YORK INSTITUTE OF STOMATOLOGY.

A MEETING of the Institute was held Tuesday evening, June 7, 1898, at the residence of Dr. C. B. Parker, No. 167 Remsen Street, Brooklyn, the President, Dr. E. A. Bogue, in the chair.

The minutes of the meeting held at the Windsor Hotel on the afternoon and evening of May 3 were read and approved.

Dr. S. E. Davenport presented a paper on "Some Properties of Vulcanite Rubber."

(For Dr. Davenport's paper, see page 579.)

### DISCUSSION.

*Dr. Davenport.*—One of the samples which were passed around, of thin vulcanized rubber, is made by the addition of new rubber to old, exactly end for end, the piece of rubber first vulcanized being filed as flat as possible on the end with a fine file, new rubber being attached to the filed end with a hot instrument and vulcanized a second time.

*The President.*—Dr. Davenport's paper is open for discussion.

*Dr. W. St. George Elliott.*—Of course, we all have our different ways of doing things, and for many years, to some extent, I have carried out this same practice; but there was a suspicion in my mind, until to-night at least, that the union has never been a very perfect one, and I have always felt called upon to use some dovetailing or its equivalent. My ordinary way is to clean the surface thoroughly and use a small wheel burr, with which I scarify the surface; in that way I get a very good union, with the addition of choloroform to get a clean surface. But I am very glad, indeed, that Dr. Davenport has undertaken these experiments, because it is a step in the direction which is so important to us as a profession. We are so inclined to the empirical; we do a thing because we are told to do it, or because it is the way we have always done it.

*Dr. J. Adams Bishop.*—I am very much interested in this line of work, and am sorry that Dr. Davenport did not say something about the vulcanizing. How is the rubber to be cured? I would like to ask Dr. Davenport if he had an upper and an under denture, and sent them to the laboratory, how he would like to have them vulcanized?

*Dr. Elliott.*—In England it is a rather common practice to vulcanize without the use of investments in the ordinary way,—that is, the rubber is fastened directly to the model by cement, then packed in anything which will keep it in form, sometimes in sand, sometimes in powdered soapstone or like material, merely to keep the parts in their respective places; it is then vulcanized. Several articles have recently appeared in the journals advocating this same method.

*Dr. A. H. Brockway.*—I was also disappointed that Dr. Davenport did not say something about the process of vulcanizing, for I am inclined to think that is something not so well understood in practice as it should be,—that is to say, many dentists fail to get as good results as they could if they knew just what to do in the matter of vulcanizing. I have not done much of that kind of work of late years, but I have done a great deal of it in the past, and I think the finest piece of vulcanite work that I ever did was the first piece. I kept the little experimental plate for a good many years, and it had a toughness and strength that I was never able to equal afterwards, and I attribute it to the fact that in my ignorance of the process I vulcanized it at a low heat and for a long time. It seems to me that I get better results in that way than I did by the usual process of vulcanizing at 320° F. for an hour or so. I have seen a good many plates that seemed to me to lack strength from some failure to properly vulcanize.

*Dr. McNaughton.*—I have been working on this thin piece, and find it has broken in two, one-third of the break being in the old crack, the rest being a new break.

*Dr. J. Adams Bishop.*—I only wish to say that vulcanite rubber, which has now been in use for more than thirty years, is one of the most useful materials that our profession has for the making of plates. I think it has a value almost as great as gold or platinum, increased according to the nicety of its preparation for the mouth. The passing of our models from the office to the laboratory is where the annoyance comes in, for there they are rushed through as rapidly as possible, with little regard to vulcanizing, cleanliness, or finish. If all of these things were carefully worked out it would make clean and healthy plates. I have seen it under all conditions, in and out of the mouth, and think more can be done with it than with any other material we use.

*Dr. F. Milton Smith.*—Dr. Davenport's paper was to me decidedly interesting. The thought occurred, as it has so many times before, why was it that for so long a time we did not appreciate the fact that new rubber if applied to clean rubber will stick there? It seems so simple when we have once tried it. The thing to know is, that the surface shall be absolutely clean and fresh; then the new rubber will cohere strictly. I do not believe it is necessary that a single mark should be made on the old rubber if it is clean and fresh. Day before yesterday I had occasion to repair a plate quickly, and I am one of those unfortunates who sometimes do a little laboratory work. My patient had two teeth extremely loose, and it was necessary to remove them. She was anxious to have new ones as soon as possible. I removed the natural teeth, and in a very few moments had made a model of the plate, had scraped it away, fitted the teeth, packed the rubber, and had it in the vulcanizing flask. It seems to me that by this method we save two-thirds of the time.

*Dr. Davenport.*—I think too much emphasis cannot be placed upon the necessity of having a perfectly clean surface, and that it must also be a dry surface, or success will not come. The old method of undercutting was absolutely necessary if the plate was to be heated and steamed, because with the moist surface there would be no cohesion unless there were undercuts; but with a dry, clean surface not a mark is necessary, as Dr. Smith has said.

Referring to the statements that nothing was said about vulcanizing, I am reminded of what Kipling says,—"That is another story."

*The President.*—I do not know that it is very far out of the line to recall the experience of one of our French friends whose vulcanizer blew up, and who had a piece of work which had been promised at a certain time. He remembered that glycerin was susceptible of being heated up to 320°, so he took his flask and put it in the glycerin and vulcanized successfully.

*Dr. C. A. Woodward.*—I have a case in practice to report, which, I think, is unique. Some four or five months ago a gentleman called upon me, who had been told that I sometimes implanted teeth. He informed me that his wife, some months before, had been taken with a severe illness, and during that time had lost her mind. Always before her illness she had been very proud of her teeth, as they were rather handsome; but after insanity came on, she became possessed with the idea that those teeth should be removed, and she set to work to get them out. Her age was about forty, and her teeth were as firm as any teeth generally found. During her early life she had lost a sixth-year molar, and between the molar and second biscuspid was a space into which she could get the handle of a spoon or fork. She finally succeeded in removing the two bicuspids. After that she would catch hold of a chair or a table, or anything that she could seize with her teeth, and pull with so much force that she broke the outer alveolar plate, so that it came out in one piece. After that, of course, it was very easy to remove the other teeth.

Upon an examination of her mouth, I saw that it was impossible to implant the teeth, owing to the loss of the alveolar process. She was extremely anxious to have her own teeth replaced in her mouth, and had the idea that it could be done, and, although I told her it was impossible to implant them, she said that I must devise some way of putting the natural teeth back.

After much thought and consultation with others, I succeeded in mounting the natural teeth upon a gold plate. I cut off the roots about one-half their length, keeping the other half to give strength, and set the natural teeth into wax as we do ordinarily, then withdrew the teeth and vulcanized the plate. After vulcanizing, I cemented the natural teeth with zinc phosphate upon the little screws in the sockets that had been made, and the accuracy and nicety with which the teeth were replaced surprised and pleased me, so accurately, indeed, that one can hardly detect that they were replaced. I may say that the teeth have been worn now about four months, and I see no reason why they should not give as much service as porcelain teeth do, and they are, of course, most natural in appearance.

I will give another little case in practice. A gentleman a week or two ago came to me with a piece of bridge-work, consisting of four incisors that he had worn for some years. Since then he had lost the bicuspids, and wished a new piece put in, with the bicuspids added to it. He was a public speaker, and it was very necessary that in removing the old piece they could be replaced again, as he could not go without them at all while making the others. Upon examination, I found that they were very firm in the mouth, and it was a query how I should take them out without destroying the bridge. I made a remark to him that I should be very lucky if I could remove the bridge without breaking one or more of the teeth. He replied, "I will take care of that part of it; you need not be alarmed about that," and said, when the bridge had been in the mouth but a short time a physician prescribed for him the vapor of listerine thrown into the mouth as hot as it could be borne and as long as he could bear it. After using the listerine, he found that his bridge-work was so loose that it nearly dropped out. I was incredulous about it, and thought it might be from some other cause. However, I told him that before he came the next time he could apply the hot listerine, and I would see what the effect was. He applied it just before coming to my office, and the bridge was so loose that he had hard work to get to the office without dropping it out. What the action of listerine is on zinc phosphate I have not had time to determine by experiment; but the bridge was very firm before applying it, and after applying it for ten or fifteen minutes the zinc phosphate was very soft and the bridge came out easily.

*Dr. Davenport.*—I wish to call attention to one phase of this case which Dr. Woodward said nothing about, and that is the determination of the gentleman who designed and carried out this most remarkable scheme that it should go through successfully. I happen to know a little something about it, as I was one of those consulted by Dr. Woodward, and, with the rest, discouraged him; and if he had taken the advice of his friends he would not have attempted it. However, his great success in New York City has probably been due more to himself than to the advice of his friends, and this remarkable success which he has exhibited to us to-night is a good illustration of that.

*Dr. Woodward.*—It is true that, upon consulting with Dr. Davenport and others, I was discouraged about my ability to vulcanize teeth. But when I get into a corner and do not know what to do, I have gone to our worthy president, Dr. Bogue. I think on that occasion he told me that to vulcanize the screws into the plates and then replace the natural teeth in the sockets with zinc phosphate would be the best thing. I carried out his suggestion, and I believe with perfect success.

*Dr. Louis C. LeRoy.*—With reference to the attachment of natural teeth to vulcanite, the only other instance which I recall is a practical case in possession of the S. S. White Dental Manufacturing Company as an exhibit of the skill of the Japanese or Chinese in the reproduction of work representing nature. They have a set of natural teeth built in a celluloid base. Of course, the supposition is that we cannot vulcanize or raise the heat of celluloid to a point to cause the same to flow about natural teeth without disintegrating them. The process as performed by Dr. Woodward is a wonderful piece of work; there seems to be a union as perfect as vulcanite would form with the natural teeth and the base upon which it is built. Dr. Woodward's work is in line with the beautiful art.

*Dr. Woodward.*—I have been told by a gentleman who has had some practice that to vulcanize natural teeth will destroy them; that when coming out of the plaster they will crumble to pieces by the heat of the vulcanizing.

*The President.*—I want to disclaim all originality. I have seen much of that work done on the other side of the water, but I do not know that I have seen anything prettier than this. Nevertheless, many natural teeth are set on vulcanite plates over there with the most beautiful results. Many of the attachments are made with sulphur, others with zinc phosphate; but I rose particularly just now to say that it seems like a reproach to all of us that, in view of the accuracy with which teeth can be fitted into a wax plate and withdrawn, a vulcanite plate made, and the teeth put back, we still continue to vulcanize on our models, always destroying them. It is getting to be rather a hobby with me to refer to a French workman who worked for me for eleven or twelve years, and who used to make his articulating models, keep the wax plate with which the bite was taken to set up his teeth upon, and bring back my plates on the articulator. I have experienced more comfort and profit from that sort of work on the other side of the water than I have experienced in New York.

*Dr. Elliott.*—Is it not the ordinary custom on the Continent as well as in England to always preserve the models?

*The President.*—It is, and we are making a constant mistake here in destroying our models. I heard a gentleman at Albany the other day speak of repairing rubber plates. He said something that was, a good deal of it, new to me. Instead of making an investment completely of plaster, he made his model for the investment by pouring in very fine plaster and shaking it out, and then for the rest of his model he poured plaster and pumice-stone, by which he said he neutralized the usual contraction of the plaster which always annoys us when we undertake to make repairs.

*Dr. Elliott.*—For many years the use of natural teeth has been more or less common; but we have always been taught that natural teeth will not answer the purpose; that they are porous and are always disagreeable, added to which it is a well-known fact that they will decay in the mouth. That has been noted over and over again. Upon one occasion I made a gold suction-plate for a Japanese official. The suction was not what it should have been. He went away and in two or three weeks he returned for me to make another, and he took out of his mouth a duplicate of my plate made of wood, copying my air-chamber and everything complete, with natural teeth inserted in the wood and held by a string. It was very beautifully done, and my gold plate he was carrying in his pocket.

*The President.*—We shall be glad to hear the report of the Committee on Operative Dentistry, of which Dr. Allan is chairman.

*Dr. Geo. S. Allan.*—In spite of the fact that the work of our committees presents many difficulties, I feel that much may be accomplished and the Institute greatly benefited. As usual with committees, the chairman has most of the work to do, but much of each chairman's effort should be directed towards interesting the other members of his committee in the work and securing their cooperation. I feel that it is fortunate for us all that my committee has secured from Dr. Raymond his promise to present some things of importance this evening.

*Dr. E. H. Raymond.*—The life-work of the dental practitioner is made up largely of saving teeth by filling them,—reconstructing those organs that have been impaired by processes of decay. Up to the present time, gold, in its varied forms, has been the standard as a filling-material. There are special cases, however, where, for the sake of appearance, general safety, and utility, solid gold fillings are not advisable. Where there is cervical decay,—that perplexing form of disintegration we so often find on the necks of the anterior teeth,—gold is not always the best material to use in checking it, as it is objectionable on account of its color. The most appropriate material for this work is porcelain. It approximates nature more closely and gives greater satisfaction, especially to our lady patients.

In 1873 I placed a section of porcelain, an inlay, on the labial surface of a superior central, for a gentleman in this city, cutting it from a plate tooth, and cementing it in with oxychloride of zinc. It proved so successful that ever since that time I have resorted to that method of filling wherever the conditions warranted it. That first section of porcelain is in the mouth to-day, but it has been reset with zinc phosphate.

About eight years ago I had occasion to treat some teeth for a young lady from the South, in whose mouth there was a marked predisposition to cervical decay. Her teeth were very white and beautifully formed. They had deep grooves along the cervical margins, which, had they been filled with gold, would have been very conspicuous, and would have spoiled the appearance of her mouth. I selected some porcelain sections, and shaping them with fine corundum stones, cemented them in with the zinc phosphate, using the rubber dam to keep them dry during the process. They are scarcely noticeable, even by her intimate friends.

In the models which are presented, it will be noticed that in one of them the decay has extended some distance beyond the gum-line. In this I have placed an inlay of porcelain gum, cutting it from a plate-tooth. A white inlay in such a cavity would make the tooth abnormally long and spoil the symmetry of the arch.

I prepare the cavities for such work in the usual way, making the undercuts a little deeper around the edges. Care must be taken not to cut too much from the centre of the cavity, for fear of injury to the pulp. The inlay should be fitted to the cavity; then, with a fine-edged stone (wheel), a slight groove should be cut around the edges of it. Roughen the under side of the inlay also, if it is thick enough to warrant it. After the color of the porcelain selected for a case is decided on, do not be disappointed if, when it is ground, fitted, and cemented in the cavity, the cement changes it to a lighter or darker shade than the tooth. The cement should be one or two shades lighter than the inlay. I usually select several colors of the oxide of zinc and mix with water, placing the inlay in the cavity with this preparation until the proper color is obtained. It takes but a moment, and is easily removed. Then mix the oxide selected with phosphoric acid. It is often necessary to mix two powders together to obtain the color desired. Enamel of porcelain being translucent and zinc phosphate opaque, one's patience will be taxed sometimes to make nature and art harmonize.

In this model will be found a tooth in which there is a large cavity. As a result, the walls are not strong, one-third of the tooth at least having disappeared. The cavity being compound,—grinding surface and proximate,—to fill it with gold would be a tedious operation. Amalgam would be very objectionable, and the non-metallic plastics would soon wear out. In such teeth I have for some years used a capping of gold plate with unusually gratifying results. Prepare the cavity as carefully as possible, leaving the edges of it strong and bevelled around the margins. Take a piece of pattern tin (25 gauge), and, after cutting it into shape, burnish it down, so as to restore the contour of the tooth. Remove this tin, and cut a duplicate in pure gold plate, same gauge; then bevelling the edges of the gold, burnish it around the edges of the cavity. It is then ready for the anchor, which may be made of any stiff metal (I use 20-carat gold), and solder it on the inside of the cap with 14-carat solder, held over a spirit lamp. Fill the cavity with soft zinc phosphate, and press the cap in place.

We have here, gentlemen, the most satisfactory method for the restoration of such frail teeth that is known, in my opinion, embodying, as it does, the advantages of the non-metallic substance inside and the durable qualities of the metallic outside. Then, too, the patient is spared the ordeal of a tedious operation, and the operator's vital force and mental balance will not suffer. The tooth, too, will last longer than if a large amount of metal were placed in it.

I use pure gold because of its malleability, and the edges can be burnished so as to make it fit the cavity accurately. Be sure to bevel the edges of the cavity. Fine stones are best for this purpose.

*Dr. Allan.*—The case which Dr. Raymond has alluded to, and which I had the pleasure of seeing, was the most perfect piece of operative work in that line I have ever seen. I had had a prejudice for many years against this inlay work, deeming it in a great measure impracticable, but when this young lady presented herself and I examined those inlays I found them almost perfect, and they could not have been detected as being artificial without the closest examination. Dr. Raymond assures us that those inlays are perfect to-day, which does him great credit, and I know the work gave the greatest possible comfort to his patient.

I wish to call attention to one fact, and that is that while he does the work practically in an off-hand manner,—that is, he cuts out his piece of tooth and grinds it roughly to a plaster model and then finishes it so as to fit the cavity,—he does it with a rapidity and certainty that I doubt are equalled by any other practitioner. I think the process takes about two hours for the usual labial cavity, and I have used more than that time on a case, and had it a total failure many a time. Dr. Raymond has placed several of these inlays in my mouth, and he did it in a business-like way,—that is to say, there was no lost time; and I have been complimented a great many times on the improved appearance of my dental organs.

The principle that he has so well demonstrated, of facing a large cavity with gold, the bulk of the filling being zinc phosphate, I think will appeal to the good judgment of all as being eminently practicable in this class of cases, saving an immense amount of energy and time. I have never practised it myself, and only judge of it by these models; but I do not see how the doctor could remove one of these facings in case there was necessity without destroying it. It seems to me that if provision were made in some way for removing the facings, it would be a very great addition to the usefulness of the operation.

*Dr. Brockway.*—Will Dr. Raymond inform us what cement he uses for these cases?

*Dr. Raymond.*—I have used almost every preparation on the market. Just now I am using a so-called German cement, sold by the Consolidated Company on Forty-second Street, N. Y.

*The President.*—How does Dr. Raymond prepare his porcelain inlays?

*Dr. Raymond.*—Some years ago I took about a pint—more or less—of porcelain teeth and broke them with a hammer. There being all shapes, sizes, and colors, I can generally select what is required from this collection. If anything special is needed, I select from the stock at the dental depots. I prefer the English teeth for many cases. They are harder to work into inlays, but they retain their color better after the cement is placed under them. By first cutting the tooth with cutting pliers one will save much grinding. Fitting these inlays is an excellent way for us to cultivate the grace of patience.

*Dr. Davenport.*—May we draw the conclusion from what Dr. Raymond says of the danger of changing the color of the porcelain inlay by the use of a cement of improper color that he might get the correct color by the use of the right cement even if the porcelain were not of the exact shade; in other words, whether the color of the finished operation did not depend quite as much upon the cement as upon the porcelain if the porcelain were translucent. Would not the English teeth be far superior to American, as in polishing down they take a much better surface than will American teeth after the enamel is ground off?

*Dr. Raymond.*—I find that after setting these inlays it is not necessary to polish the surface highly. As long as the surface is moist one cannot tell whether it is polished or not.

*Dr. Kimball.*—Dr. Raymond spoke of cutting the edges with a fine wheel. May I ask what he meant by that?

*Dr. Raymond.*—After the inlay is shaped to the cavity, I take a fine corundum wheel and make a little groove all around the edge of it. The cavity being dovetailed, the cement then holds the inlay in securely. I have seldom known one to come out.

*Dr. George Evans.*—Take an approximal cavity, which would consume about two hours to excavate, and fill with gold-foil. If Dr. Raymond would undertake to fill that cavity with porcelain and finish it as he explains, how long would it probably take him?

*Dr. Raymond.*—I never attempt to use porcelain inlays in approximal cavities, unless there is plenty of room. One cannot make a success of it except under very favorable circumstances.

The time required to place an inlay depends entirely on the conditions,—the size, position, and shape of the cavity. Having had considerable experience, I can work them in almost as rapidly as I can put in gold fillings. The question of dexterity is largely one of practice in this line of work.

*Dr. Elliott.*—I would say, in regard to the last subject, that the process of inlaying is carried on much more largely in England than here. Mr. Dall, of Scotland, prepared the extended apparatus alluded to; it is sold by Ash, but is largely confined to round and half-round cavities. To me it is altogether a new feature that English teeth are less translucent, because I have always understood that their teeth were the most translucent, and for that reason I preferred them. The difference in manufacture is that in the English goods the ingredients are ground together and then poured into the moulds, while the American teeth are made in a thick paste and forced into the mould; the result is that in the English teeth the particles are much finer than in the American. Dr. de Trey is an old Swiss friend of mine who has been experimenting with gold for twenty years. There are, of course, two kinds of gold; foil and precipitate, the main distinctive feature between the different precipitates of gold is the mode of manufacture. The de Trey gold, as I understand it, consists merely in so controlling the electric current that the deposit of crystals is exceedingly slow and fine, and the result is that you get a mass of crystal gold, not in the form of a mass which is to be subsequently cut up, but in the form of a sheet generally about an inch square, the sheet being about as thick as ordinary blotting-paper, and the crystals are apparently deposited upon a sheet of this foil. Now it seems to me that the relative value of the precipitated gold is in its physical character. I think we would get much the same result that we do from de Trey's gold with any other sponge-gold that is prepared in the same way in thin sheets, were it not that the crystals of de Trey gold are so minute. After experimenting with this gold for some years, having imported, I believe, the first lot into this country, I now use it almost exclusively. Suffice it to say that, in my opinion, de Trey's gold is going to largely take the place of other forms of gold, and for this reason: for a given amount of force, one gets greater condensation. That is practically the secret of the whole thing. In order to test the matter I have made some experiments which I found very interesting. I made a detachable steel mould and filled it in different ways so that the result was a bar of gold. I then tested this for solidity by the specific gravity test. My impression has always been that, theoretically and mechanically, the best fillings were made by the solidification of parallel layers. Flat foil, when used with the hand-mallet gave me a specific gravity of 15 +, pure gold being 19 +. I used the de Trey gold, with a view of testing its use with the R. A. engine-mallet and a very small point. It gave a specific gravity of not over 9, it being too much chopped up. I then took the de Trey gold and put it in by hand-pressure; I got a specific gravity of 11 +. This morning I put in a filling of the same gold with the hand-mallet in the ordinary way, and it gave me a specific gravity of within a fraction of 17. The bar put in with a small point broke very readily, it having apparently no cohesive strength.[1]

I have made a number of instruments to use with the de Trey gold, which all may wish to examine. There is nothing about them

[1] The bars used to test for specific gravity were afterwards broken with the following result:

Average of seven breaks, Watts's gold hand-mallet, five pounds, approximately.

Flat No. 4 globe-foil hand-mallet, sixteen pounds, approximately.

De Trey gold hand-pressure, seven pounds, approximately.

De Trey gold hand-mallet, four and a half pounds, approximately.

De Trey gold R. A. engine-mallet one and a half pounds, approximately.

All bars were the same size and were de Trey's $\frac{122}{1000}$ inch broad (over $\frac{1}{10}$) by $\frac{69}{1000}$ (a little over $\frac{1}{16}$) inch thick.

Result. For strength, flat foil is the best; for adaptation, facility, and density, de Trey's.

except that they have bulbous extremities. Operators should be cautious about one thing; in all cavities, in the use of this gold, direct pressure should be had in all directions. With the ordinary hand-pressure a specific gravity of 11 may be secured, which gives good surface hardness. With hand-pressure also, pressure in any direction can be gained, whereas with malleting, it is far more complicated.

*The President.*—I think that the committees have given us this evening a large repast, and I am sorry that time could not be given to discuss, for instance, the gold spoken of by Dr. Elliott. Dr. Elliott has not brought up the point of deepest interest,—namely, the adaptability of the gold, of which he speaks, to the edges and walls of cavities; he speaks of specific gravity, but that is only one quality, and not by any manner of means the most important. As long as Dr. Elliott has brought up the subject in the manner in which he has, showing his familiarity with it, I hope he will go a little further at one of our meetings in the fall, and bring to our attention other points, upon which he has not touched. He is probably better qualified to do so than any other one of us, and will bring out a discussion that may be of real importance to all.

*Dr. Allan.*—If this report of the Committee on Operative Dentistry could be continued at one of the fall meetings, this subject of the de Trey gold could be more fully brought up and discussed. I have a package of instruments here, but there is not time to exhibit them, and there are many points to bring out on this important subject.

There is another preparation of the crystal gold series just designed by the S. S. White Company, some of which I tried to get to show this evening, but I was unable to secure it.

*The President.*—I can only thank the committee for its earnest efforts to bring something before us of interest and importance, and I trust the Executive Committee will take into consideration what Dr. Allan has requested for the fall meeting, so far as is consistent with other arrangements. If I may allude to another thing, our friend from Plymouth, Dr. Shumway, showed us some experiments in regard to the cohesive qualities of gold, and in the course of those experiments he remarked that he gets absolutely solid gold in his cavities by wiping gold onto gold or tin with light ivory points. These are ideas worth bringing out by a self-constituted or other committee that shall experiment in that direction and find out the actual facts.

*Dr. Allan.*—Before moving to adjourn, I would suggest that the

Institute pass a vote of thanks to Dr. Woodward and Dr. Woodward's patient for their kindness in allowing us to see that beautiful piece of work. The patient has been put to much personal inconvenience and shows her interest in the subject in a graceful and kindly way.

Adjourned.

S. E. Davenport, D.D.S., M.D.S.,
*Editor The New York Institute of Stomatology.*

